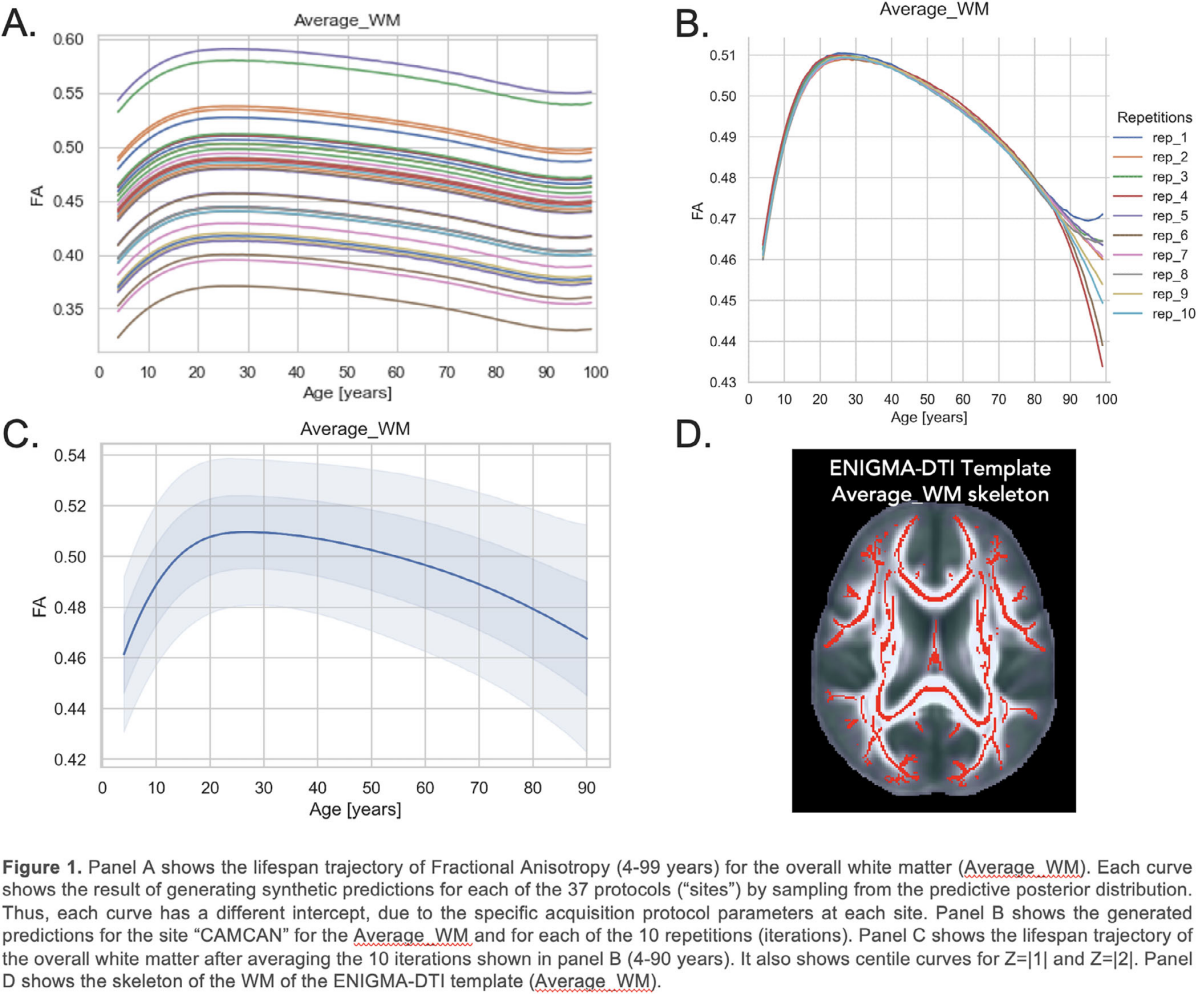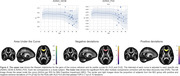# Large‐scale Normative Modeling of Brain Microstructure over the Human Lifespan

**DOI:** 10.1002/alz.093445

**Published:** 2025-01-09

**Authors:** Julio E Villalon‐Reina, Alyssa H Zhu, Sebastian M Benavidez, Clara Moreau, Yixue Feng, Emily Laltoo, Tamoghna Chattopadhyay, Elnaz Nourollahimoghadam, Sophia I Thomopoulos, Leila Nabulsi, Katherine E Lawrence, Michael W Weiner, Clifford R. Jack, Talia M Nir, Neda Jahanshad, Paul M. Thompson

**Affiliations:** ^1^ Imaging Genetics Center, Mark and Mary Stevens Neuroimaging & Informatics Institute, University of Southern California, Marina del Rey, CA USA; ^2^ University of Southern California, Los Angeles, CA USA; ^3^ Imaging Genetics Center, Mark and Mary Stevens Neuroimaging & Informatics Institute, Keck School of Medicine, University of Southern California, Marina del Rey, CA USA; ^4^ Imaging Genetics Center, Mark and Mary Stevens Neuroimaging and Informatics Institute, Keck School of Medicine, University of Southern California, Marina del Rey, CA USA; ^5^ University of California San Francisco (UCSF), San Francisco, CA USA; ^6^ Department of Radiology, Mayo Clinic, Rochester, MN USA

## Abstract

**Background:**

Normative models (NM) of brain metrics based on large, diverse populations offer novel strategies to detect individual brain abnormalities. To create an age‐dependent statistical model of brain microstructure over the human lifespan, we built the largest multi‐site NM of white matter (WM) diffusion tensor imaging (DTI) metrics based on 54,591 subjects. We used state‐of‐the‐art tools to adjust for site‐dependent effects.

**Method:**

We used Hierarchical Bayesian Regression (HBR) to determine the age trajectory and lifespan centile curves (ages 3‐100 years) by merging data from different sections of the lifespan. We analyzed 19 international public datasets with the ENIGMA‐DTI protocol. Mean fractional anisotropy (FA), mean, axial, and radial diffusivity (MD, AxD, RD) were extracted for 21 bilateral ROIs from the JHU‐WM atlas and the whole WM skeleton. Regressions were run with each metric per ROI as a function of age and sex, and the DTI protocol as the batch effect (37 protocols). Z‐scores were derived for 81 patients with Alzheimer’s disease (AD; mean age: 77y±8.4, 46M/35F) and 225 MCI participants (mean age: 75.1y±8.1, 127M/98F) and extreme deviations (Z>|2|) were quantified for each condition. ROI‐wise areas under the ROC curve were calculated to determine the classification accuracy of the Z‐scores.

**Result:**

The average FA for the overall WM skeleton reached its peak value at 27 years, with minima for MD, RD and AxD at 47, 42 and 54 years, respectively. We found extreme FA deviations (Z>2) for MCI (AUC=0.67) and AD (AUC=0.66) in the *genu* of the corpus callosum, extreme RD deviations (Z<2) in the *tapetum* (AUC=0.73) and Extreme AxD deviations (Z>2) for MCI in the *s*uperior longitudinal fasciculus (AUC=0.65). See Figures 1 & 2.

**Conclusion:**

NM of brain microstructure with HBR yielded lifespan trajectories for widely‐used DTI metrics. By pooling large scale multi‐site dMRI to create a statistical reference, single subject deviations are detectable across the lifespan for AD and MCI. These NM will be publicly available to the research community.